# U.S. public perceptions on whether risk of dementia and stroke can be modified through maintaining or changing lifestyle

**DOI:** 10.1186/s12889-025-25077-x

**Published:** 2025-11-28

**Authors:** Jasper R. Senff, Mark Jun Shah-Ostrowski, Reinier W.P. Tack, Courtney Nunley, Caroline R. Palys, Sharon Ng, Akashleena Mallick, Leidys Gutierrez-Martinez, Jonathan Duskin, Tamara N. Kimball, Savvina Prapiadou, Sandro Marini, Evy Reinders, Katelin Sherman, Ayneisha Tinoble, H. Bart Brouwers, Setareh Akhavan, Amytis Towfighi, Cyprien A. Rivier, Guido J. Falcone, Kevin Sheth, Ronald M. Lazar, Sarah Ibrahim, Aleksandra Pikula, Zeina Chemali, Cornelia van Duijn, Gregory Fricchione, Rudolph E. Tanzi, Nirupama Yechoor, Christopher D. Anderson, Valerie Purdie Greenaway, Koen B. Pouwels, Jonathan Rosand, Sanjula Dhillon Singh

**Affiliations:** 1 Brain Care Labs, Department of Neurology, Massachusetts General Brigham, Boston, MA USA; 2 McCance Center for Brain Health, Department of Neurology, Massachusetts General Hospital, Boston, MA USA; 3 Broad Institute of MIT and Harvard, Cambridge, MA USA; 4 Center for Genomic Medicine, Massachusetts General Hospital, Boston, MA USA; 5 Department of Neurology and Neurosurgery, Brain Center Rudolf Magnus, University Medical Center Utrecht, Utrecht, The Netherlands; 6 Nuffield Department of Population Health, University of Oxford, Oxford, UK; 7 Department of Psychology, Columbia University, New York, NY USA; 8 LA County Department of Health Services, Los Angeles, CA USA; 9 Department of Neurology, University of Southern California, Los Angeles, CA USA; 10 Department of Neurology, Yale School of Medicine, New Haven, CT USA; 11 Yale Center for Brain and Mind Health, Yale School of Medicine, New Haven, CT USA; 12 MCKnight Brain Institute, Department of Neurology, School of Medicine, University of Alabama, Birmingham, AL USA; 13 Program for Health System and Technology Evaluation, Department of Neurology, Toronto General Hospital Research Institute, Toronto Western Hospital, Toronto, ONT Canada; 14 Centre for Advancing Collaborative Healthcare & Education (CACHE), University of Toronto, Toronto, ON Canada; 15 The Jay and Sari Sonshine Centre for Stroke Prevention & Cerebrovascular Brain Health, Krembil Brain Institute, 16, Toronto, ONT Canada; 16 Jay and Sari Sonshine Centre for Stroke Prevention and Cerebrovascular Brain Health, Krembil Brain Institute, University Health Network, Toronto, ONT Canada; 17 Lawrence S Bloomberg Faculty of Nursing, University of Toronto, Toronto, ONT Canada

## Abstract

**Background:**

Epidemiological studies suggest that approximately 40% of dementia and 60% of stroke cases could be prevented through adequate control of modifiable risk factors. Limited data are available on the public perceptions in the United States (U.S.) on whether the risk of dementia and stroke can be modified through lifestyle changes.

**Methods:**

A survey utilizing questions from validated questionnaires was distributed to a sample of the general U.S. population. We performed multivariable logistic regression analyses for which the binary exposure was ever having known someone with dementia or stroke, and the primary outcomes were the perceptions on whether dementia and stroke risk could be modified through maintaining or changing lifestyle.

**Results:**

We included 1,478 participants (mean [SD] age: 45.5 [15.9], 51.1% female), of whom 80% (*N* = 1185) ever knew someone with dementia or stroke. Over 75% of all participants perceived that a healthy lifestyle can lower dementia and stroke risks. Following multivariable analyses, participants who ever knew someone with dementia or stroke were more likely to agree that maintaining (adjusted Odds Ratio [aOR] = 1.41, 95%CI:1. 10–1.96) and that changing lifestyle (aOR = 1.59, 95%CI:1.14–2.24) reduces dementia risk, when adjusted for age, sex assigned at birth, race/ethnicity, level of education, employment status, and being a caregiver for someone with dementia or stroke. Participants who knew someone with dementia or stroke were also more likely to agree that maintaining (aOR = 1.77, 95%CI:1.27–2.47) or changing lifestyle (aOR = 2.31, 95%CI:1.41–3.76) reduces stroke risk when adjusted for similar confounders.

**Discussion:**

This cross-sectional cohort, mimicking the general U.S. population, demonstrated that over 80% of individuals ever knew someone with dementia or stroke and that this was positively associated with the perceptions that dementia and stroke risk could be modified through lifestyle changes. The widespread exposure of the U.S. public to dementia and stroke first-hand can be leveraged into more effective preventive strategies.

**Supplementary Information:**

The online version contains supplementary material available at 10.1186/s12889-025-25077-x.

## Introduction

 The *Brain Health Crisis* encompasses millions of people worldwide who suffer from age-related non-communicable brain diseases (including dementia and stroke) [1]. In 2019, 50 million people were living with dementia and 101 million with stroke globally, with expected numbers of 150 million cases of dementia and >200 million cases of stroke by 2050 [2]. Epidemiological studies suggest that approximately 45% of all dementia cases [3] and at least 60% of all stroke cases [4] are attributable to modifiable risk factors and, therefore, could be in part prevented through adequate risk factor control. Additionally, dementia and stroke have a partly shared underlying pathophysiology of neurodegeneration and cerebrovascular disease [5], resulting in an overlap of many modifiable risk factors, including hypertension, cholesterol, blood sugar, dietary habits, body mass index (BMI), physical activity, alcohol, and smoking [3–5].

The perception that dementia and stroke risk could be modified through lifestyle changes is essential for people making actual behavioral changes that address the modifiable risk factors that have been shown to be associated with a risk reduction of dementia and stroke [6]. Health education tailored to the characteristics of individuals (age, sex assigned at birth, race/ethnicity, education, and experience with the disease) has shown to be more effective than general education in achieving behavioral change [7, 8]. Those who have experience with age-related brain disease, such as dementia, through knowing someone or being a caregiver, have a higher level of health literacy and perception of preventability [9, 10]. While acknowledging that the mere perception of preventability is not sufficient to achieve behavioral changes, it serves as the critical first step towards people making actual behavioral changes [6, 7]. There is no data available from a cohort that mimics the general U.S. population on the perceptions whether lifestyle changes could modify the risk of dementia and stroke. Previous studies showed a limited perception whether the risk of dementia (44% agreed, *N* = 590) [11] and stroke (68–69% agreed, *N* = 877) [12, 13] can be modified through lifestyle, with substantial differences in perceptions among age groups, sex assigned at birth, racial/ethical groups, geographical location, and having experience with dementia or stroke [9, 11, 14, 15].

We, therefore, aimed to conduct a survey to assess the perceptions in a cohort that mimics the U.S. general population on whether lifestyle changes could modify the risk of dementia and stroke, as well as to identify any gaps and differences between subgroups in these perceptions. We hypothesized that people who ever had known somebody with dementia or stroke would be more likely to perceive that lifestyle changes could modify the risks of dementia and stroke.

## Methods

### Survey development

A literature review (Table S1) was conducted to inform and guide the selection of previously validated questions to include in our questionnaire. The final survey, entitled the Harvard-Oxford Brain Care Awareness Survey (HOBCAS) (Table S2), was a combination of (i) demographic questions from the *‘American Community Survey’*, specifically adapted for the U.S. population [16], (ii) questions on preventability and modifiable risk factors for dementia and stroke from the ‘*Stroke Knowledge Assessment Tool’* (SKAT) [17] and *‘Dementia Knowledge Assessment Tool’* (DKAT) [18], (iii) questions on motivation, facilitators and barriers for behavior change from the *‘Motivation to Change Lifestyle and Health Behaviors for Dementia Risk Reduction Scale* [19], and (iv) questions from the *‘Brain Care Score* (a scientifically validated tool developed to empower people worldwide to make lifestyle changes that are associated with a lower risk of dementia and stroke) [20]. We dichotomized or presented the questions on a Likert scale ranging from 1 to 5, as appropriate [21]. Finally, aiming to provide an appropriate reading level for the general U.S. population, we asked questions that required a reading level of 7th to 8th grade on average [22]. This was achieved with a Flesh-Kincaid Grade level of 8.4 and SMOG Index of 8.1. We also allowed participants to provide qualitative feedback on any of the questions after taking the HOBCAS.

### Distribution

The HOBCAS survey was distributed to the general U.S. population, specifically requesting a representative cohort, utilizing the vendor platform Prolific. In short, the Prolific platform is designed to enable researchers to access non-convenient, representative samples by employing sophisticated targeting capabilities, as previously described [23, 24].

### Statistical analyses

#### Descriptive analyses

Discrete variables were reported as counts and proportions, while continuous variables were reported as means and standard deviations (SD) or, if non-normally distributed, medians and interquartile ranges (IQR). Normal distributions were assessed using Shapiro-Wilk tests. Continuous data were analyzed using Student T-tests for normal distributions and Mann-Whitney U tests for non-normal distributions. Chi-square or Fisher Exact tests were assessed for discrete variables. We presented all exact responses in the corresponding supplementary tables to minimize data loss and maximize transparency. The baseline demographics of participants were compared to the most recent (2022) U.S. census data to ensure a nationally representative cohort [25]. The sample was weighted for demographics that significantly differed from the U.S. census data, using a raking method utilized through the ‘*anesrake’* package in Rstudio Version 3.3.0 [26]. In line with previously published research [27, 28], this method adjusts the influence of each respondent so that the overall sample more closely resembles the U.S. population on key demographics, such as age, race/ethnicity, education, marital status, and employment. For the weighting, we categorized age through three groups (< 40, 40–60, >60) [9], race/ethnicity through five groups (Non-Hispanic White, Non-Hispanic Black/African American, Asian, Hispanic/Latino, and Other), and level of education through three groups (maximum high school, associate degree/bachelor’s degree and graduate degree/master’s degree or higher) [25]. We analyzed four questions that addressed the perceptions whether maintaining a healthy lifestyle can reduce dementia and stroke risk (general questions), and whether changing lifestyle and health habits can reduce the participant’s own risk of dementia and stroke (personal questions) (Questions 21, 27, 35, and 36 [Table S2]).

#### Primary analyses

We performed univariable and multivariable logistic regression models with our main outcome of having the perceptions that lifestyle could change the risk of dementia and stroke. We combined “somewhat agreeing” and “strongly agreeing” equaling agreeing, and “strongly disagreeing”, “somewhat disagreeing” and “not sure” equaling “not agreeing”. If the outcome contained a negative (Question 27: Maintaining a healthy life does not reduce a person’s risk of having a stroke) we combined “somewhat disagreeing” and “strongly disagreeing” equaling disagreeing, while “somewhat agreeing”, “strongly agreeing” and “not sure” equaling agreeing [9, 14, 29]. Our main exposure was ever having known somebody with dementia or stroke. This exposure was selected a-priori based on our pre-defined hypothesis and prior literature suggesting that people who have ever known someone with dementia or stroke would be more likely to perceive that lifestyle changes could modify the risks of dementia and stroke [9, 14, 30]. For the multivariable analyses, we based our choice of confounders on previous epidemiological data [9, 11, 14, 15] and statistical testing.

#### Secondary analyses

In addition, we performed secondary univariable logistic regression analyses to examine the associations between four additional exposures including (i) age, (ii) sex assigned at birth, (iii) race/ethnicity, (iv) education levels with the aforementioned outcomes – to assess gaps and differences in these perceptions in subgroups of the general U.S. population. These four exposures were selected based on previously published data [9, 11, 14, 15]. We refrained from performing multivariable analyses for all four additional exposures to limit the number of statistical tests performed and decrease risks of type 1 error [31]. As we only found consistent significant differences between sex assigned at birth and dementia outcomes in our univariable analyses, we performed exploratory multivariable logistic regression to assess these associations with the addition of confounding factors [9, 11, 14, 15].

### Sensitivity analyses

We performed four sensitivity analyses to test for the robustness of our results. First, we assessed if there would be any differences if we used ‘ever having been a caregiver for someone with stroke or dementia’ as an exposure rather than ‘knowing somebody with dementia or stroke’. Second, we removed employment status from the model that aimed to assess the associations between ‘knowing somebody with dementia or stroke’ and our outcomes, as employment status could be a mediating factor rather than a confounder (on the causal pathway) of these associations. Third, we re-ran the primary outcomes models after excluding all participants who responded “not sure” and dichotomized the remaining responses into “agreeing” and “disagreeing.”. Fourth, to further assess the robustness of our findings, we performed a sensitivity analysis evaluating inconsistent responses to similarly themed but oppositely worded questions: Q17 (“Dementia cannot be prevented by changing lifestyle behavior”) and Q21 (“Maintaining a healthy lifestyle reduces the risk of developing dementia”). Inconsistency was defined as providing opposite responses after reverse-coding the answers to Q17.

We presented all our results as odds ratios (OR) following univariable logistic regression analyses or adjusted OR (aOR) following multivariable logistic regression analyses with corresponding 95% confidence intervals (CI) and p-values. We analyzed selected questions important for the research objectives of this study. A p-value of < 0.05 was considered statistically significant. Statistical analyses were performed using Rstudio Version 3.3.0 and IBM SPSS Statistics 29.0.1.

## Results

### Cohort characteristics

Our cohort comprised of 1,478 participants from the U.S. general population (98.5% response rate) with a mean age of 45.5 years (SD = 15.9), of which 51.1% were female and 74.8% Non-Hispanic White (Table 1). When comparing the demographic data of our participants with the most recent U.S. Census data [25], we found differences in age, race/ethnicity, level of education, marital status, and employment status and weighted our cohort correspondingly (Table S3).

**Table 1:** Cohort characteristics (*N* = 1478)

		Unweighted	Weighted	Missing *N*=
Age (mean, SD)		45.47 (15.9)	43 (16.9)	9 (0.6%)
Female (n,%)		754 (51.8)	733 (50.4)	14 (0.9%)
Race/ethnicity (n,%)	Non-Hispanic White	1106 (75.3)	887 (60.9)	10 (0.7%)
	Non-Hispanic Black/African American	192 (13.0)	178 (12.2)	
	Asian	91 (6.2)	72 (4.9)	
	Hispanic/Latino/Spanish	62 (4.2)	277 (19.0)	
	Other	17 (1.2)	44 (3.0)	
Education (n,%)	High School Diploma or less	453 (30.7)	531 (36.5)	17 (1.2%)
	Associates degree	713 (48.2)	720 (49.5)	
	Graduate Degree	295 (20.0)	203 (14)	
Marital status (n,%)	Never married	611 (41.3)	487 (34.3)	43 (2.9%)
	Currently married	603 (40.8)	683 (48.0)	
	Separated	20 (1.4)	24 (1.7)	
	Divorced	161 (10.9)	149 (10.5)	
	Widowed	40 (2.7)	78 (5.5)	
Employment (n,%)	Employed	1008 (69.9)	876 (60.8)	36 (2.4%)
	Unemployed	156 (10.8)	39 (2.7)	
	Not in labor force	278 (19.3)	526 (36.5)	
Living situation (n,%)	I do not have a steady place to live	9 (0.6)	15 (1.1)	23 (1.6%)
	I have a steady place to live but I am worried about losing it	160 (10.8)	144 (10.0)	
	I have a steady place to live	1286 (87.0)	1282 (89.0)	
Living status (n,%)	Homeowner	767 (51.9)	737 (50.9)	22 (1.5%)
	Renting	514 (34.8)	525 (36.3)	
	Occupying without payment of cash rent	163 (11.0)	169 (11.7)	
	Other	12 (0.8)	17 (1.2)	
House income (n,%)	$ 0 - $ 20.550	164 (11.1)	156 (10.7)	9 (0.6%)
	$ 20.551 - $ 83.550	746 (50.5)	739 (50.5)	
	$ 83.551 - $ 178.150	437 (29.6)	456 (31.2)	
	$ 178.151 - $ 340.100	105 (7.1)	98 (6.7)	
	$ >340.101 -	17 (1.1)	13 (0.9)	
Personal income (n,%)	$ 0 - $ 10.275	319 (21.6)	329 (22.3)	4 (0.3%)
	$ 10.276 - $ 41.775	492 (33.3)	514 (34.9)	
	$41.776 - $89.075	455 (30.8)	431 (29.2)	
	$ 89.076 - $170.050	169 (11.4)	176 (11.9)	
	$ 170.051- $215.950	21 (1.4)	14 (1.0)	
	$ 215.951 - $539.900	15 (1.0)	8 (0.5)	
	≥ $539.901	3 (0.2)	2 (0.1)	
Experience with dementia/stroke	Do you know someone with dementia/stroke	1198 (81.1)	1185 (80.2)	1 (0.1%)
	Caregiver for dementia/stroke	388 (26.3)	398 (26.9)	0 (0)

Baseline characteristics, presented both unweighted as obtained from the questionnaire and weighted. *Abbreviations*: *N* number of participants, *SD* Standard deviation % the percentage

### Descriptive analyses

Of all participants, 80% (*N* = 1185, 95%CI: 78%−82%) had ever known someone with dementia or stroke, and 27% (*N* = 398, 95%CI: 25%−29%) had ever been caregivers for someone with dementia or stroke (Table 1). Of all our participants, 76% of participants (*N* = 1117, 95%CI: 74%−78%) agreed with the statement that “maintaining a healthy lifestyle reduces the risk of developing dementia”, and 78% of participants (*N* = 1146, 95%CI: 76%−80%) agreed with the statement that “changing lifestyle and health habits can reduce their risk of dementia”. Of all our participants, 76% of participants (*N* = 1127, 95%CI: 74%−79%) disagreed with the statements that “maintaining a healthy lifestyle does not reduce a person’s risk of stroke”, and 92% of participants (*N* = 1361, 95%CI: 91%−94%) agreed with the statement that “changing lifestyle and health habits can reduce their risk of having a stroke” (Table S4).

#### Dementia risk

We found that 76% (*N* = 904, 95% CI: 74%−79%) of participants who had ever known someone with dementia or stroke perceived that maintaining a healthy lifestyle could reduce dementia risk, compared to 73% (*N* = 212, 95%CI: 67%−78%) amongst those who had not. We found that 79% (*N* = 931, 95%CI: 75%−81%) of participants who ever had known someone with dementia or stroke perceived that changing lifestyle could reduce their dementia risk, compared to 73% (*N* = 213, 95%CI: 67%−79%) amongst those who had not.

#### Stroke risk

We found that 78% (*N* = 925, 95%CI: 76%−81%) of participants who had ever known someone with dementia or stroke perceived that maintaining a healthy lifestyle reduces stroke risk, compared to 69% (*N* = 201, 95%CI: 64%−74%) amongst those who had not. We found that 94% (*N* = 1107, 95%CI: 92%−95%) of participants who ever had known someone with dementia or stroke perceived that changing lifestyle reduces their stroke risk compared to 87% (*N* = 254, 95%CI: 83%−91%) amongst those who had not.

### Primary analyses: univariable and multivariable logistic regression models with primary outcomes and the exposure ‘having ever known somebody with dementia or stroke’

**Fig. 1:**
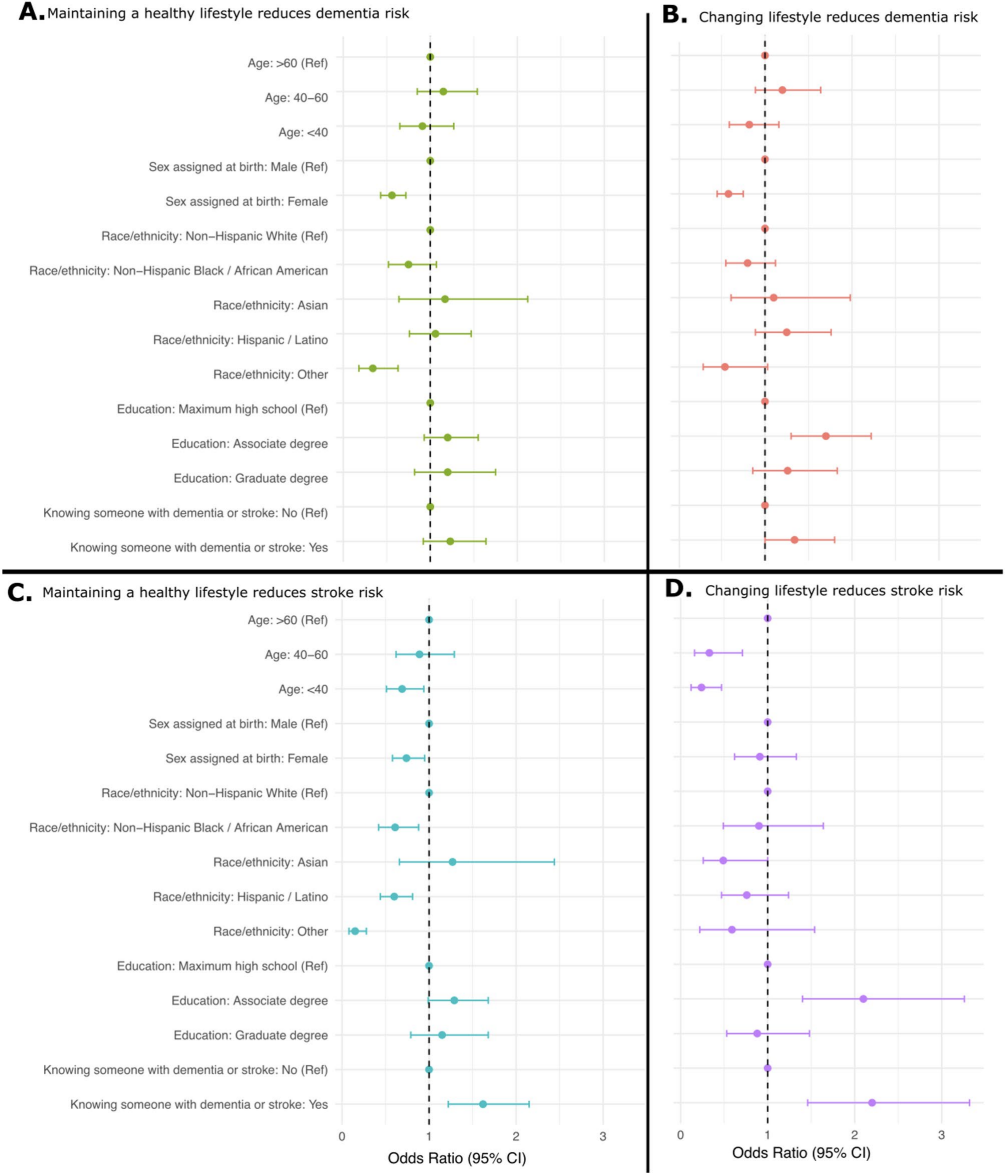
Univariable logistic analysis for the four primary outcomes. **A** Agreeing that maintaining a healthy lifestyle reduces the risk of developing dementia. **B** Agreeing that changing lifestyle and health habits can help reduce my risk of developing dementia. **C** Disagreeing that maintaining a healthy lifestyle does not reduce the risk of developing stroke. **D** Agreeing that changing lifestyle and health habits can help reduce my risk of having a stroke. Abbreviations Ref. Reference category. CI. Confidence interval

In univariable logistic regression analyses, we found that participants who had ever known someone with dementia or stroke were more likely to perceive that maintaining a healthy lifestyle reduces the risk of developing stroke (OR:1.62, [95%CI:1.22–2.15], *P* < 0.001), and more likely to perceive that changing lifestyle health habits can reduce their the risk of having a stroke (OR:2.20 [95%CI:1.46–3.22], *P* < 0.001). In univariable logistic regression analyses for dementia risk, we found no significant differences (Fig. 1, Table S5).

**Fig. 2:**
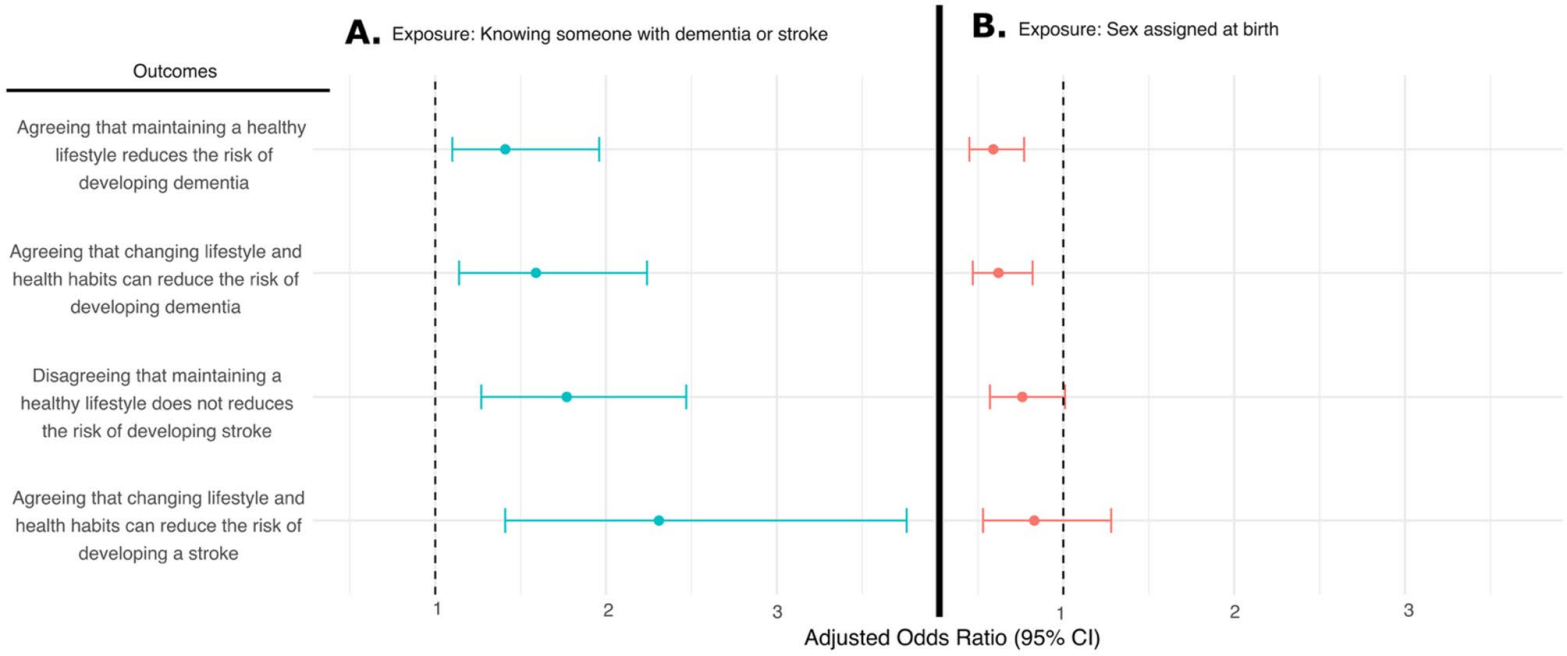
Multivariable logistic regression for the four primary outcomes.** A.** Primary analysis. Exposure: Knowing someone with dementia or stroke (Reference: Not knowing someone with dementia or stroke). Adjusted for age, sex assigned at birth, race/ethnicity, level of education, knowing someone with dementia or stroke, marital status, employment status and being a caregiver for someone with dementia or stroke. **B. **Secondary Analysis. Exposure: Sex assigned at birth - Female (Reference: Male). Adjusted for age, race/ethnicity, level of education, knowing someone with dementia or stroke, marital staus, employment status and total personal income. Abbreviations: CI: Confidence Interval

Following multivariable regression analyses, adjusted for age, sex assigned at birth, race/ethnicity, level of education, marital status, employment status, and ever having been a caregiver for someone with dementia or stroke, participants who had ever known someone with dementia or stroke were more likely to agree that maintaining (adjusted Odds Ratio [aOR] = 1.41, 95%CI:1.10–1.96) and that changing lifestyle or health habits (aOR = 1.59, 95%CI:1.14–2.24) reduces dementia risk compared to those who had not. Participants who had ever known someone with dementia or stroke were more likely to disagree that maintaining a healthy lifestyle does not reduces the risk of developing stroke (aOR = 1.77, 95%CI:1.27–2.47) and more likely to agree that changing lifestyle and health habits can reduce their risk of developing stroke (aOR: 2.31, 95% CI:1.41–3.76) **(**Fig. 2, Table, S6**)**.

### Secondary analyses

In univariable logistic regression analyses, we found that women, compared to men, were less likely to perceive that maintaining a healthy lifestyle reduces dementia risk (OR:0.56, [95%CI:0.43–0.72]) and less likely to perceive that changing lifestyle can help reduce the risk of developing dementia (OR:0.58 [95%CI: 0.45–0.75]). Women, as compared to men, were also less likely to perceive that maintaining a healthy lifestyle reduces the risk of developing stroke (OR: 0.74, [95%CI:0.58–0.95]). We found no consistent differences in the primary outcomes for the exposures age, race/ethnicity, and education levels – only a potential trend that older age, those with higher education levels, and those of Non-Hispanic White race/ethnicity were more likely to perceive that maintaining or changing lifestyle could reduce the risks of dementia and stroke (Fig. 1, Table S5).

In multivariable logistic regression analyses: women, compared to men, were less likely to perceive that maintaining a healthy lifestyle reduces dementia risk (aOR: 0.59 [95%CI: 0.45–0.77], *P* < 0.001) and were less likely to perceive that changing lifestyle and health habits reduce dementia risk (aOR: 0.62 [95%CI: 0.47–0.82], *P* < 0.001) when adjusted for age, race/ethnicity, level of education, knowing someone with dementia or stroke, marital status, employment status and total personal income. We did not find statistically significant differences between women and men with respect to the perceptions of whether the risk of stroke can be reduced through lifestyle **(**Fig. 2, Table S6,7**)**.

### Sensitivity analyses

In sensitivity analyses, we found no differences in associations between ever having known someone with stroke or dementia and ever having been a caregiver for someone with stroke or dementia with any of the four outcomes (Table S8). When employment status was removed as a confounder from our models, we found no differences in association in three of our main models, with one model becoming borderline significant (Table S9). The sensitivity analysis excluding “not sure” responses yielded similar results to the main models (Table S10). When assessing inconsistent responses, we found that a total of 17.7% of participants gave opposite responses to Q17 and Q21.

## Discussion

We present the first data on the perceptions whether lifestyle changes can change the risks of dementia and stroke in a cohort that mimics the general U.S. population. We found that 80% of participants had ever known someone with dementia or stroke and that these participants were more likely to perceive that maintaining or changing their lifestyle could change the risks of dementia and stroke compared to those who had not. In secondary analyses, we found that women, compared to men, were less likely to perceive that dementia risk can be reduced through a healthy lifestyle but that there was no difference in the perceptions of stroke risk.

### Differences in perceptions based on prespecified subgroups

At least two-thirds of all participants perceived that the risk of dementia and stroke could be reduced by changing or maintaining a healthy lifestyle. Our finding was that participants who ever had known someone with dementia or stroke were more likely to perceive that a healthy lifestyle reduces dementia and stroke risk, aligned with previous literature from other countries than the U.S [14, 32]. For all our statistical analyses which investigated the perceptions whether lifestyle can reduce dementia or stroke risks, participants more often perceived that lifestyle could modify the risk of stroke in comparison to the risk of dementia, aligning with previous literature as well [11, 29, 33]. Furthermore, we found that women were less likely to perceive that dementia is preventable through maintaining or changing to a healthy lifestyle. Two previous studies described a comparison based on sex assigned at birth and the perception of preventability of dementia: with one study showing no difference between women and men [11], and one study showed similar results as in our cohort [15]. Additionally, we found inconsistent results in perceptions based on other subgroups (age, race/ethnicity, and educational level), similar to previous studies [9, 15, 33]. We found that 18% of participants gave opposing responses to Q17 and Q21, similar to findings from other questionnaire-based studies [34, 35].While these questions are conceptually related, they are not identical—Q17 addresses beliefs about complete prevention, whereas Q21 reflects beliefs about risk reduction, which could contribute to the differences observed.

### Strengths and weaknesses

Our study’s strengths include providing the first data on the perceptions whether dementia and stroke risk can be reduced through lifestyle in a cross-sectional cohort that mimics the general U.S. population. We utilized the most recent U.S. census data to weigh the responses, ensuring the most representative survey results possible [25]. Furthermore, we used the Prolific Online Platform, which provides high-quality data for survey distribution and data acquisition, including a 98.5% response rate and low rates of missing data (less than 3% for baseline characteristics) [24]. We provided the participants with a feasible survey with a reading level for 7th to 8th grade and allowed for qualitative feedback - with none of the participants providing negative feedback on any of the questions addressed in this manuscript [22].The limitations of our current study include that we administered questions online, whilst most questionnaires used to build the HOBCAS were validated face-to-face or on paper [17–19]. However, the current literature is inconclusive about whether this results in bias, with most experts not expecting substantial biases [24]. Utilizing the vendor platform Prolific limited the study population to those with internet access and the ability to complete a 62-question survey, which could have affected the external generalizability of our findings. We were unable to verify the reliability of our obtained data through the Prolific platform. Past research suggested that online questionnaires’ accuracy is high and not influenced by survey length [36]. Furthermore, data provided by Prolific has demonstrated high quality, characterized by participants’ attentiveness, comprehensive instructions, and reliable answers [24]. Importantly, there were differences between our survey compared to the 2022 Census data in demographics, including education, employment, and a lack of Hispanic/Latino participants. To improve generalizability, we weighted our sample based on the 2022 U.S. census data to approach or mimic the general U.S. population to the best of our abilities [26–28]. We specifically selected demographics from the U.S. Census data to weigh the data and mimic it, thereby limiting us to other important demographics, such as cohabitation. The cross-sectional design of the survey does not allow for making any statement about causality, with the exposure and outcome assessed at the same time point. Furthermore, we restricted to closed questions for feasibility and user-friendliness purposes, which may have led to overestimating positive responses compared to surveys that use open-ended questions, as described in previously [9, 21, 33]. We chose a 5-point Likert scale, in line with previously published literature [9, 14, 29], to allow for descriptive analyses to show more detailed and nuanced replies from our participants. We opted for binary logistic regression models over ordinal- or multinomial models. While this decision led to some data loss during analyses, this approach was driven by small counts in essential categories coupled with significant variability in beta coefficients, undermining the proportional odds assumption for ordinal models. Furthermore, binary logistic regression was chosen to maintain the interpretability of results in a pragmatic context, avoiding the complexities associated with numerous coefficients [21, 37]. Further, the inconsistency in the phrasing of the outcome—using “agreeing” for one outcome and “disagreeing” for another—may have introduced interpretive variability. We recognize this as a limitation and will harmonize the outcome phrasing in future iterations of the HOBCAS questionnaire to ensure more accurate, comparable responses. Lastly, concerning our secondary analyses, while we found potential trends that older age, those with higher education levels, and those of Non-Hispanic White ethnicity/race perceived that maintaining or changing lifestyle could change their risk of dementia or stroke we did not further explore these participant characteristics in regard to the outcomes. Future research should include investigating these associations in well-powered population-based cohorts.

### Implication and future directions

This is the first study that presents data on the perceptions of whether dementia and stroke risk can be modified through maintaining or changing lifestyle, in a cohort that mimics the general U.S. population. Research has shown that people’s perceptions of the potential for lifestyle to change the risk of diseases are critically important for making actual behavioral changes [11, 29]. In addition, health education tailored to the characteristics of individuals has proven to be more effective than general education in order to achieve actual behavioral changes [6, 7]. In line with recent publications from the American Heart Association and the American Academy of Neurology: more data is warranted on these perceptions [38, 39]. As our study identified subgroups of the U.S. population that could be more likely to perceive that lifestyle changes can modify the risks of dementia and stroke: further research needs to be conducted in large population-based cohorts in the U.S., after which tailored health education materials could be designed and prioritised for these subgroups (including males, ethnicities/races other than Non Hispanic Whites, those with lower level of education and those aged younger than 60). Previous literature shows that individuals with less education also have lower knowledge of stroke and dementia risk factors, and these risk factors are more prevalent in this group [40]—making them a key population with high preventive potential for lifestyle change. Future directions include performing a validation study using a representative prospective cohort study to confirm our findings. Additionally, future research projects could separate our main exposure, knowing someone with stroke or dementia, and assess associations with the outcomes. This research could subsequently explore the participant’s perceptions of the associations between dementia and stroke. For now, the results of this study may be leveraged for future research studies that focus on the perceptions in these subgroups, as well as for health education implementation policies and prioritization aiming to prevent dementia and stroke in the U.S.

In summary, this study shows that most individuals in a cohort mimicking the general U.S. population perceived that the risk of dementia and stroke can be reduced through lifestyle changes. More than 80% knew someone affected by these conditions, and this experience was positively associated with the perception of preventability. This widespread exposure to dementia and stroke may be leveraged to design more effective prevention strategies.

## Supplementary Information

Supplementary Material 1

## Data Availability

Data is provided within the manuscript or supplementary information files. Data is avaliable upon reasonable request through contact with the corresponding author.
