# Supplementary material for: U.S. public perceptions on whether risk of dementia and stroke can be modified through maintaining or changing lifestyle
Source: BMC Public Health. 2025 Nov 28;25:4191. doi: 10.1186/s12889-025-25077-x (PMC12664128; doi:10.1186/s12889-025-25077-x)
Supplement: Supplementary file 1 — Supplementary Material 1 [file 12889_2025_25077_MOESM1_ESM.docx]

**U.S. public perceptions on Whether Risk of Dementia and Stroke Can Be Modified Through Maintaining or Changing Lifestyle**

Jasper R. Senff, MD^1-5^; Mark Jun Shah-Ostrowski, BSc^6^; Reinier W.P. Tack, MD^1-5^; Courtney Nunley ^1^; Caroline R. Palys^1,7^;

Sharon Ng BSc^1-4^; Akashleena Mallick, MD^1-4^; Leidys Gutierrez-Martinez, MD MSc^1-4^; Jonathan Duskin MD^1-4,^; Tamara N.

Kimball MD^1-4^; Savvina Prapiadou MD^1-4^; Sandro Marini MD^1-4^;Evy Reinders MD^1-4^; Katelin Sherman BSc^1-4^; Ayneisha

Tinoble^1-4^; H. Bart Brouwers MD^5^ PhD; Setareh Akhavan, MPH^8-9^;Amytis Towfighi MD^8-9^; Cyprien A. Rivier MD^10-11^; Guido

J Falcone MD ScD^10-11^; Kevin Sheth MD PhD^10-11^; Ronald M. Lazar PhD^12^; Sarah Ibrahim RN PhD^13-15^; Aleksandra Pikula

MD^16-18^; Zeina Chemali MD MPH^2^, Cornelia van Duijn PhD FMedSci^6^; Gregory Fricchione MD, PhD^1,19^; Rudolph E. Tanzi

PhD^2^; Nirupama Yechoor MD^1-4^; Christopher D. Anderson MD MMsc^1-4,^; Valerie Purdie Greenaway PhD^7^; Koen B. Pouwels PhD^6^; Jonathan Rosand, MD MSc^1-4^; Sanjula Dhillon Singh, MD PhD MSc^1-4^

**Affiliations:**

1. Brain Care Labs, Department of Neurology, Massachusetts General Brigham, Boston, MA, USA
2. McCance Center for Brain Health, Department of Neurology, Massachusetts General Hospital, Boston, MA, USA
3. Broad Institute of MIT and Harvard, Cambridge, MA, USA
4. Center for Genomic Medicine, Massachusetts General Hospital, Boston, MA, USA
5. Department of Neurology and Neurosurgery, Brain Center Rudolf Magnus, University Medical Center Utrecht, Utrecht, The Netherlands
6. Nuffield Department of Population Health, University of Oxford, Oxford, United Kingdom
7. Department of Psychology, Columbia University, New York, NY, USA
8. LA County Department of Health Services, Los Angeles, CA, USA
9. Department of Neurology, University of Southern California, Los Angeles, CA, USA
10. Department of Neurology, Yale School of Medicine, New Haven, CT, USA
11. Yale Center for Brain and Mind Health, Yale School of Medicine, New Haven, CT, USA
12. MCKnight Brain Institute, Department of Neurology, School of Medicine, University of Alabama, Birmingham, AL USA
13. Program for Health System and Technology Evaluation; Toronto General Hospital Research Institute, Department of Neurology, Toronto Western Hospital,Toronto, ONT, Canada
14. Centre for Advancing Collaborative Healthcare & Education (CACHE), University of Toronto, Toronto, ON, Canada
15. The Jay and Sari Sonshine Centre for Stroke Prevention & Cerebrovascular Brain Health
16. Krembil Brain Institute, Toronto, ONT, Canada
17. Jay and Sari Sonshine Centre for Stroke Prevention and Cerebrovascular Brain Health, Krembil Brain Institute, University Health Network, Toronto, ONT, Canada
18. Lawrence S Bloomberg Faculty of Nursing, University of Toronto, Toronto, ONT, Canada

**Corresponding author:**

- Sanjula Dhillon Singh
- Email address: ssingh32@mgh.harvard.edu
- Address: McCance Center for Brain Health, Massachusetts General Hospital, Harvard Medical School, Revolution Drive 399, Sommerville, 02145 MA, United States of America

No. of tables: 3 (8 supplementary)

**Supplementary Tables**

| **Table S1.** Search syntax literature search for risk factor identification | | |
| --- | --- | --- |
| **#** | **Search query Pubmed** | **Results** |
| 1 | "knowledg*"[All Fields] OR "percep*"[All Fields] OR "awareness"[MeSH Terms] OR "awareness"[All Fields] OR "aware"[All Fields] OR "awarenesses"[All Fields] OR "litera*"[All Fields] OR "educat*"[All Fields] Sort by: Most Recent  "knowledg*"[All Fields] OR "percep*"[All Fields] OR "awareness"[MeSH Terms] OR "awareness"[All Fields] OR "aware"[All Fields] OR "awarenesses"[All Fields] OR "litera*"[All Fields] OR "educat*"[All Fields] | 4,410,181 |
| 2 | "risk*"[All Fields] OR (("risk"[MeSH Terms] OR "risk"[All Fields]) AND "factor*"[All Fields]) OR "factor*"[All Fields] | 8,318,841 |
| 3 | "dementi*"[All Fields] OR "stroke*"[All Fields] OR ("cerebrovascular disorders"[MeSH Terms] OR ("cerebrovascular"[All Fields] AND "disorders"[All Fields]) OR "cerebrovascular disorders"[All Fields]) OR ("cerebral small vessel diseases"[MeSH Terms] OR ("cerebral"[All Fields] AND "small"[All Fields] AND "vessel"[All Fields] AND "diseases"[All Fields]) OR "cerebral small vessel diseases"[All Fields] OR ("cerebral"[All Fields] AND "small"[All Fields] AND "vessel"[All Fields] AND "disease"[All Fields]) OR "cerebral small vessel disease"[All Fields]) | 822,637 |
| 4 | "surve*"[Title] OR "question*"[Title] OR "assessment"[Title] OR "evaluation*"[Title] | 1,228,142 |
| 5 | #1 AND #2 AND #3 AND #4 | 2,528 |
|  | **Criteria** |  |
| Inclusion | 1. Survey and/ or questionnaires. 2. Describing (a) knowledge, (b) perceptions and or awareness of modifiable risk factors for stroke and/or dementia |  |
| Exclusion | 1. Disease specific populations (e.g.: stroke survivors) 2. Medically trained populations (e.g.: medical students or nurses) |  |

| **Table S2 HOBCAS Questionnaire** | | | | |  |
| --- | --- | --- | --- | --- | --- |
| **No.** | **Question** | | | **Options** | **Data used in this manuscript** |
|  | Please enter your unique Prolific ID | | | Open question | Yes |
|  | Please enter your 5-number zip code | | | Open question | No |
|  | Please enter your age | | | Open Question | Yes |
|  | Please select the race/ethnicity with which you identify | | | a. White  b. Black / African American  c. American Indian / Alaskan Native  d. Hispanic / Latino / Spanish Origin  e. Chinese  f. Filipino  g. Asian Indian  h. Other Asian  i. Vietnamese  j. Korean  k. Japanese  l. Native Hawaiian  m. Samoan  n. Chamorro  o. Other Pacific Islander  p. Other | Yes |
|  | What was your biological sex assigned at birth? | | | a. Female  b. Male  c. Intersex  d. None of the above describe me  e. Prefer not to answer  f. Unknown | Yes |
|  | Please select the highest level of education you have completed | | | a. No schooling completed  b. Nursery-Kindergarten  c. Grade 1-12: No diploma  d. High School diploma, GED or alternative credential  e. Associate’s degree or bachelor’s degree  f. Graduate degree | Yes |
|  | Which of the following categories best describes your marital status | | | a. Currently married  b. Divorced  c. Separated  d. Widowed  e. Never married | Yes |
|  | Which of the following categories best describes your employment status? | | | a. Full-time employment  b. Part-time employment  c. Self-employed  d. Student  e. Full-time caregiver  f. Not employed, looking for work  g. Not employed, NOT looking for work  h. Retired  i. Unable to work | Yes |
|  | What is your living situation today? | | | a. I have a steady place to live  b. I have a steady place to live today,   but am worried about losing it in the future  c. I do not have a steady place to live | Yes |
|  | Which of the following best describes your living status? | | | a. Homeowner  b. Renting  c. Occupying without payment of cash rent  d. Other | Yes |
|  | Total household income: how much total combined money did all members of your household earn in 2022? This includes money from jobs, net income from businesses, farms or rent; pensions; dividends; interest; social security payments and any other income received by members of your household. Please report the total amount of money earned - do not subtract the amount you paid in taxes or any deductions listed on your tax returns. | | | a. $0-$20,550  b. $20,551-$83,550  c. $83,551-$178,150  d. $178,151-$340,100  e. $340,101-$431,900  f. $431,901-$647,850  g. $647,851 or more | Yes |
|  | Total personal income: how much money did you earn personally in 2022? This includes money from jobs, net income from businesses, farms or rent; pensions; dividends; interest; social security payments and any other income received by you. Please report the total amount of money earned - do not subtract the amount you paid in taxes or any deductions listed on your tax returns. | | | a. $0-$10,275  b. $10,276-$41,775  c. $41,776-$89,075  d. $89,076-$170,050  e. $170,051-$215,950  f. $215,951-$539,900  g. $539,001 or more | Yes |
|  | Have you ever known someone who has had a diagnosis of dementia or stroke? | | | a. Yes  b. No | Yes |
|  | Have you ever been a caregiver for someone who has had a diagnosis of dementia or stroke? | | | a. Yes  b. No | Yes |
|  | Dementia is a normal part of the ageing process | | | 5-point Likert scale: (i) strongly disagree, (ii) somewhat disagree, (iii) not sure, (iv) somewhat agree or (v) strongly agree | No |
|  | Alzheimer's disease is the most common form of dementia | | | 5-point Likert scale: (i) strongly disagree, (ii) somewhat disagree, (iii) not sure, (iv) somewhat agree or (v) strongly agree | No |
|  | Dementia cannot be prevented by changing lifestyle behavior | | | 5-point Likert scale: (i) strongly disagree, (ii) somewhat disagree, (iii) not sure, (iv) somewhat agree or (v) strongly agree | No |
|  | Having a high blood pressure does not increase a person's risk of developing dementia | | | 5-point Likert scale: (i) strongly disagree, (ii) somewhat disagree, (iii) not sure, (iv) somewhat agree or (v) strongly agree | No |
|  | Dementia can be diagnosed at very early stages | | | 5-point Likert scale: (i) strongly disagree, (ii) somewhat disagree, (iii) not sure, (iv) somewhat agree or (v) strongly agree | No |
|  | Dementia can be caused by blood vessel disease | | | 5-point Likert scale: (i) strongly disagree, (ii) somewhat disagree, (iii) not sure, (iv) somewhat agree or (v) strongly agree | No |
|  | Maintaining a healthy lifestyle reduces the risk of developing dementia | | | 5-point Likert scale: (i) strongly disagree, (ii) somewhat disagree, (iii) not sure, (iv) somewhat agree or (v) strongly agree | Yes |
|  | Exercise is generally beneficial for reducing the risk of dementia | | | 5-point Likert scale: (i) strongly disagree, (ii) somewhat disagree, (iii) not sure, (iv) somewhat agree or (v) strongly agree | No |
|  | Medications are the most effective way to reduce the risk of developing dementia | | | 5-point Likert scale: (i) strongly disagree, (ii) somewhat disagree, (iii) not sure, (iv) somewhat agree or (v) strongly agree | No |
|  | Smoking is a risk factor for stroke and dementia | | | 5-point Likert scale: (i) strongly disagree, (ii) somewhat disagree, (iii) not sure, (iv) somewhat agree or (v) strongly agree | No |
|  | Stroke is more common in women | | | 5-point Likert scale: (i) strongly disagree, (ii) somewhat disagree, (iii) not sure, (iv) somewhat agree or (v) strongly agree | No |
|  | Having a stroke increases a person's risk of having a second stroke at a later time | | | 5-point Likert scale: (i) strongly disagree, (ii) somewhat disagree, (iii) not sure, (iv) somewhat agree or (v) strongly agree | No |
|  | Maintaining a healthy lifestyle does not reduce a person's risk of stroke | | | 5-point Likert scale: (i) strongly disagree, (ii) somewhat disagree, (iii) not sure, (iv) somewhat agree or (v) strongly agree | Yes |
|  | Having a high cholesterol does not increase your risk of stroke | | | 5-point Likert scale: (i) strongly disagree, (ii) somewhat disagree, (iii) not sure, (iv) somewhat agree or (v) strongly agree | No |
|  | Drinking small amounts of alcohol (a glass of wine a day) is protective against stroke and dementia | | | 5-point Likert scale: (i) strongly disagree, (ii) somewhat disagree, (iii) not sure, (iv) somewhat agree or (v) strongly agree | No |
|  | Less than 7 hours of sleep, or an untreated sleep disorder can be a risk factor for dementia | | | 5-point Likert scale: (i) strongly disagree, (ii) somewhat disagree, (iii) not sure, (iv) somewhat agree or (v) strongly agree | No |
|  | I feel at high risk of developing dementia | | | 5-point Likert scale: (i) strongly disagree, (ii) somewhat disagree, (iii) not sure, (iv) somewhat agree or (v) strongly agree | No |
|  | I feel at high risk of having a stroke | | | 5-point Likert scale: (i) strongly disagree, (ii) somewhat disagree, (iii) not sure, (iv) somewhat agree or (v) strongly agree | No |
|  | Information and advice from medical professionals can help me reduce my risk of developing dementia | | | 5-point Likert scale: (i) strongly disagree, (ii) somewhat disagree, (iii) not sure, (iv) somewhat agree or (v) strongly agree | No |
|  | Information and advice from medical professionals can help me reduce my risk of having a stroke | | | 5-point Likert scale: (i) strongly disagree, (ii) somewhat disagree, (iii) not sure, (iv) somewhat agree or (v) strongly agree | No |
|  | Changing lifestyle and health habits can help reduce my risk of developing dementia | | | 5-point Likert scale: (i) strongly disagree, (ii) somewhat disagree, (iii) not sure, (iv) somewhat agree or (v) strongly agree | Yes |
|  | Changing lifestyle and health habits can help reduce my risk of having a stroke | | | 5-point Likert scale: (i) strongly disagree, (ii) somewhat disagree, (iii) not sure, (iv) somewhat agree or (v) strongly agree | Yes |
|  | Changing lifestyle and health habits is difficult to maintain over a long period of time | | | 5-point Likert scale: (i) strongly disagree, (ii) somewhat disagree, (iii) not sure, (iv) somewhat agree or (v) strongly agree | No |
|  | I am too busy to change my lifestyle and health habits | | | 5-point Likert scale: (i) strongly disagree, (ii) somewhat disagree, (iii) not sure, (iv) somewhat agree or (v) strongly agree | No |
|  | My financial situation does not allow me to change my lifestyle and behavior | | | 5-point Likert scale: (i) strongly disagree, (ii) somewhat disagree, (iii) not sure, (iv) somewhat agree or (v) strongly agree | No |
|  | Learning more about dementia and stroke motivates me to change my lifestyle | | | 5-point Likert scale: (i) strongly disagree, (ii) somewhat disagree, (iii) not sure, (iv) somewhat agree or (v) strongly agree | No |
|  | Having risk factors for dementia and stroke makes me want to change my lifestyle | | | 5-point Likert scale: (i) strongly disagree, (ii) somewhat disagree, (iii) not sure, (iv) somewhat agree or (v) strongly agree | No |
|  | I am confident that I can change my lifestyle and behaviour so I can reduce the risk of developing dementia and stroke | | | 5-point Likert scale: (i) strongly disagree, (ii) somewhat disagree, (iii) not sure, (iv) somewhat agree or (v) strongly agree | No |
|  | To what extent do you believe these factors contribute to making your brain healthy? | Maintaining a healthy blood pressure | | 5-point Likert scale: (i) very useful, (ii) somewhat useful, (iii) not sure, (iv) not very useful, (v) not useful at all | No |
| **43.** |  | Maintaining healthy blood sugar levels | | 5-point Likert scale: (i) very useful, (ii) somewhat useful, (iii) not sure, (iv) not very useful, (v) not useful at all | No |
| **43.** |  | Maintaining healthy levels of cholesterol | | 5-point Likert scale: (i) very useful, (ii) somewhat useful, (iii) not sure, (iv) not very useful, (v) not useful at all | No |
| **43.** |  | Maintaining a healthy weight | | 5-point Likert scale: (i) very useful, (ii) somewhat useful, (iii) not sure, (iv) not very useful, (v) not useful at all | No |
| **43.** |  | Maintaining a healthy diet | | 5-point Likert scale: (i) very useful, (ii) somewhat useful, (iii) not sure, (iv) not very useful, (v) not useful at all | No |
| **43.** |  | Low alcohol intake | | 5-point Likert scale: (i) very useful, (ii) somewhat useful, (iii) not sure, (iv) not very useful, (v) not useful at all | No |
| **43.** |  | Not smoking | | 5-point Likert scale: (i) very useful, (ii) somewhat useful, (iii) not sure, (iv) not very useful, (v) not useful at all | No |
| **43.** |  | Consistent physical activity | | 5-point Likert scale: (i) very useful, (ii) somewhat useful, (iii) not sure, (iv) not very useful, (v) not useful at all | No |
| **43.** |  | A healthy sleep schedule | | 5-point Likert scale: (i) very useful, (ii) somewhat useful, (iii) not sure, (iv) not very useful, (v) not useful at all | No |
| **43.** |  | Managing levels of stress | | 5-point Likert scale: (i) very useful, (ii) somewhat useful, (iii) not sure, (iv) not very useful, (v) not useful at all | No |
| **43.** |  | Maintaining close social relationships | | 5-point Likert scale: (i) very useful, (ii) somewhat useful, (iii) not sure, (iv) not very useful, (v) not useful at all | No |
| **43.** |  | Finding purpose / meaning in life | | 5-point Likert scale: (i) very useful, (ii) somewhat useful, (iii) not sure, (iv) not very useful, (v) not useful at all | No |
| **43.** |  | Brain training apps and games | | 5-point Likert scale: (i) very useful, (ii) somewhat useful, (iii) not sure, (iv) not very useful, (v) not useful at all | No |
| **43.** |  | Brain supplements (fish oil, etc.) | | 5-point Likert scale: (i) very useful, (ii) somewhat useful, (iii) not sure, (iv) not very useful, (v) not useful at all | No |
| **43.** |  | Practicing meditation and mindfulness techniques | | 5-point Likert scale: (i) very useful, (ii) somewhat useful, (iii) not sure, (iv) not very useful, (v) not useful at all | No |
| **44.** | Which of these factors do you believe are the most difficult to change/maintain in daily life? | Maintaining a healthy blood pressure | | 5-point Likert scale: (i) very useful, (ii) somewhat useful, (iii) not sure, (iv) not very useful, (v) not useful at all | No |
| **44.** |  | Maintaining healthy blood sugar levels | | 5-point Likert scale: (i) very useful, (ii) somewhat useful, (iii) not sure, (iv) not very useful, (v) not useful at all | No |
| **44.** |  | Maintaining healthy levels of cholesterol | | 5-point Likert scale: (i) very useful, (ii) somewhat useful, (iii) not sure, (iv) not very useful, (v) not useful at all | No |
| **44.** |  | Maintaining a healthy weight | | 5-point Likert scale: (i) very useful, (ii) somewhat useful, (iii) not sure, (iv) not very useful, (v) not useful at all | No |
| **44.** |  | Maintaining a healthy diet | | 5-point Likert scale: (i) very useful, (ii) somewhat useful, (iii) not sure, (iv) not very useful, (v) not useful at all | No |
| **44.** |  | Low alcohol intake | | 5-point Likert scale: (i) very useful, (ii) somewhat useful, (iii) not sure, (iv) not very useful, (v) not useful at all | No |
| **44.** |  | Not smoking | | 5-point Likert scale: (i) very useful, (ii) somewhat useful, (iii) not sure, (iv) not very useful, (v) not useful at all | No |
| **44.** |  | Consistent physical activity | | 5-point Likert scale: (i) very useful, (ii) somewhat useful, (iii) not sure, (iv) not very useful, (v) not useful at all | No |
| **44.** |  | A healthy sleep schedule | | 5-point Likert scale: (i) very useful, (ii) somewhat useful, (iii) not sure, (iv) not very useful, (v) not useful at all | No |
| **44.** |  | Managing levels of stress | | 5-point Likert scale: (i) very useful, (ii) somewhat useful, (iii) not sure, (iv) not very useful, (v) not useful at all | No |
| **44.** |  | Maintaining close social relationships | | 5-point Likert scale: (i) very useful, (ii) somewhat useful, (iii) not sure, (iv) not very useful, (v) not useful at all | No |
| **44.** |  | Finding purpose / meaning in life | | 5-point Likert scale: (i) very useful, (ii) somewhat useful, (iii) not sure, (iv) not very useful, (v) not useful at all | No |
| **44.** |  | Brain training apps and games | | 5-point Likert scale: (i) very useful, (ii) somewhat useful, (iii) not sure, (iv) not very useful, (v) not useful at all | No |
| **44.** |  | Brain supplements (fish oil, etc.) | | 5-point Likert scale: (i) very useful, (ii) somewhat useful, (iii) not sure, (iv) not very useful, (v) not useful at all | No |
| **44.** |  | Practicing meditation and mindfulness techniques | | 5-point Likert scale: (i) very useful, (ii) somewhat useful, (iii) not sure, (iv) not very useful, (v) not useful at all | No |
| **45.** | If you had to pick only one, which of the following factors do you believe has the greatest impact on keeping your brain healthy? | | | a. Maintaining a healthy blood pressure  b. Maintaining healthy blood sugar levels  c. Maintaining healthy levels of cholesterol  d. Maintaining a healthy weight  e. Maintaining a healthy diet  f. Low alcohol intake  g. Not smoking  h. Consistent physical activity  i. A healthy sleep schedule  j. Managing levels of stress  k. Maintaining close social relationships  l. Finding purpose / meaning in life | No |
| **46** | Please rank the following factors based on which you would be most likely to change in your own daily life | | | a. Maintaining a healthy blood pressure  b. Maintaining healthy blood sugar levels  c. Maintaining healthy levels of cholesterol  d. Maintaining a healthy weight  e. Maintaining a healthy diet  f. Low alcohol intake  g. Not smoking  h. Consistent physical activity  i. A healthy sleep schedule  j. Managing levels of stress  k. Maintaining close social relationships  l. Finding purpose / meaning in life | No |
| **47** | Have you ever been diagnosed with hypertension (high blood pressure)? If you know your most recent blood pressure measurement, please enter it below using the "other" text option | | | a. Yes  b. No  c. Other | No |
| **48** | Have you ever been diagnosed with diabetes? If you know your most recent Hemoglobin A1c (HbA1c) measurement, please enter it below using the "other" text option | | | a. Yes, I have a diagnosis of diabetes  b. I have a diagnosis of prediabetes (impaired glucose tolerance)  c. No  d. Other | No |
| **49** | Have you ever been diagnosed with hypercholesterolemia (high cholesterol)? | | | a. Yes, and I am on medication  b. Yes, but I am not on medication  c. no | No |
| **50** | Please enter your current weight in pounds and ounces (lbs and oz) | | |  | No |
| **51** | Please enter your height in feet and inches (ft and in) | | |  | No |
| **52** | Which of the following do you include in your typical diet? | | | a. 4.5 servings of fruit and vegetables per day  b. 2 servings of lean protein per day  c. 3 or more servings of whole grains per day  d. Less than 1,5 grams of sodium per day  e. Less than 36oz of sugar sweet beverages (soda, juice etc.) per week | No |
| **53** | How many alcoholic drinks do you consume per week? | | | a. 0-1 alcoholic drinks per week  b. 2-3 alcoholic drinks per week  c. 4 or more alcoholic drinks per week | No |
| **54** | Do you smoke? | | | a. Current smoker  b. Quit within the last year  c. Quit more than one year ago  d. Never smoked | No |
| **55** | How much aerobic exercise do you do per week? | | | a. Less than 150 minutes of moderate, or less than 75 minutes of high intensity physical activity per week  b. At least 150 minutes of moderate, or more than 75 minutes of high intensity physical activity per week | No |
| **56** | How long do you sleep every night? | | | a. Less than 5 hours per night  b. 5-7 hours per night  c. More than 7 hours per night | No |
| **57** | How would you describe your daily level of stress? | | | a. High level of stress than often makes it difficult to function  b. Moderate level of stress that occasionally makes it difficult to function  c. Manageable level of stress that rarely makes it difficult to function | No |
| **58** | How many close social connections do you have? Close social connections are people other than your partner or children, that you feel close with and could talk about private matters or call upon for help. | | | a. I have few or no close connections other than my partner or children  b. I have at least two people, other than my partner or children, that I feel close with and could talk about private matters or call upon for help | No |
| **59** | Do you often struggle to find meaning in life? | | | a. I often struggle to find value or purpose in my life  b. I generally feel that my life has meaning and/or purpose | No |
| **60** | Who do you trust when you are looking for information about your health? | | Primary care physicians | 5-point Likert scale: (i) strongly trust, (ii) somewhat trust(iii) not sure, (iv) somewhat do not trust (v) strongly do not trust | No |
| **60** |  |  | Specialist physicians | 5-point Likert scale: (i) strongly trust, (ii) somewhat trust(iii) not sure, (iv) somewhat do not trust (v) strongly do not trust | No |
| **60** |  |  | Other healthcare professionals (nurses, NP's) | 5-point Likert scale: (i) strongly trust, (ii) somewhat trust(iii) not sure, (iv) somewhat do not trust (v) strongly do not trust | No |
| **60** |  |  | Religious organizations and leaders | 5-point Likert scale: (i) strongly trust, (ii) somewhat trust(iii) not sure, (iv) somewhat do not trust (v) strongly do not trust | No |
| **60** |  |  | Scientists | 5-point Likert scale: (i) strongly trust, (ii) somewhat trust(iii) not sure, (iv) somewhat do not trust (v) strongly do not trust | No |
| **60** |  |  | Podcasts, radio and television | 5-point Likert scale: (i) strongly trust, (ii) somewhat trust(iii) not sure, (iv) somewhat do not trust (v) strongly do not trust | No |
| **60** |  |  | Family and friends | 5-point Likert scale: (i) strongly trust, (ii) somewhat trust(iii) not sure, (iv) somewhat do not trust (v) strongly do not trust | No |
| **60** |  |  | Social media | 5-point Likert scale: (i) strongly trust, (ii) somewhat trust(iii) not sure, (iv) somewhat do not trust (v) strongly do not trust | No |
| **60** |  |  | Newspapers and magazines (including online editions) | 5-point Likert scale: (i) strongly trust, (ii) somewhat trust(iii) not sure, (iv) somewhat do not trust (v) strongly do not trust | No |
| **60** |  |  | Websites, or self-search (Google) | 5-point Likert scale: (i) strongly trust, (ii) somewhat trust(iii) not sure, (iv) somewhat do not trust (v) strongly do not trust | No |
| **60** |  |  | Politicians | 5-point Likert scale: (i) strongly trust, (ii) somewhat trust(iii) not sure, (iv) somewhat do not trust (v) strongly do not trust | No |
| **60** |  |  | Government health agencies | 5-point Likert scale: (i) strongly trust, (ii) somewhat trust(iii) not sure, (iv) somewhat do not trust (v) strongly do not trust | No |
| **60** |  |  | Charitable organizations | 5-point Likert scale: (i) strongly trust, (ii) somewhat trust(iii) not sure, (iv) somewhat do not trust (v) strongly do not trust | No |
| **61** | Did you understand all of the questions you were asked in this survey? If no, please write down the question numbers below. | | | Open | Yes |
| **62** | Would you have preferred to take this survey in a language other than English? | | | Open | Yes |

**.**

| **Table S3 Baseline characteristics compared with US census 2022 data** | | | | |
| --- | --- | --- | --- | --- |
| **Characteristics** | | **HOBCAS** | **Census** | **P - value** |
| Age (mean, SD) |  | 45.47 (15.9±) | 39.0 ± 0.1 | **<0.001** |
| Age, n (%) | <40 years | 602 (40.7%) | 171,060,408 (51.3%) |  |
|  | 40-60 years | 514 (34.8%) | 83,029,320 (24.9%) |  |
|  | >60 years | 362 (24.5%) | 79,361,359 (23.8%) |  |
| Female, n (%) |  | 754 (51.0%) | 168,059,348 (50.4%) | 0.663 |
| Race/ethnicity, n (%) | Non-Hispanic White | 1106 (74.8%) | 202,889,017 (60.87%) | **<0.001** |
|  | Non- Hispanic Black / African American | 192 (13.0%) | 40,603,656 (12.18%) |  |
|  | Asian | 91 (6.2%) | 16,132,837 (4.85%) |  |
|  | Hispanic / Latino | 62 (4.2%) | 63,553,639 (19.07%) |  |
|  | Other | 17 (1.2%) | 9,235,033 (3.03%) |  |
| Education , n (%) (only participants ≥25 years) | Less than high school | 8 (0.6%) | 23,889,542 (10.4%) | **<0.001** |
|  | High School Diploma | 341 (26.7%) | 59,953,562(26.1%) |  |
|  | Associates degree | 634 (49.6%) | 113,705,033(49.5%) |  |
|  | Graduate Degree | 280 (21.9%) | 32,158,999 (14.0%) |  |
| Marital status, n (%) | Never married | 611 (41.3%) | 93,961,020 (34.3%) | **<0.001** |
|  | Currently married | 603 (40.8%) | 131,490.640 (48.0%) |  |
|  | Separated | 20 (1.4%) | 4,656,960 (1.7%) |  |
|  | Divorced | 161 (10.9%) | 28,763,577 (10.5%) |  |
|  | Widowed | 40 (2.7%) | 13,833,911 (5.5%) |  |
| Employment, n (%) | Employment | 1008 (69.9%) | 163,889,633 (60.8%) | **<0.001** |
|  | Unemployment | 156 (10.8%) | 7,277,994 (2.7%) |  |
|  | Not in labor force | 278 (19.3%) | 98,387,691 (36.5%) |  |
| House owners, n (%) | Owner occupier housing | 930 (64.4%) | 84,675,845 (65.2%) | 0.654 |
|  | Renting occupeer housing | 514 (35.6%) | 45,195,082(34.8%) |  |

**Legend.** Table S3. Baseline characteristics compared with US census 2022 data. Abbreviations: SD:

Standard deviation. N: Number of participants. %: percentage.

| **Table S4 Perceptions on preventability of dementia and stroke** | | | | | | |  |
| --- | --- | --- | --- | --- | --- | --- | --- |
| Statements on dementia N, (%) | Strongly disagree | Somewhat disagree | Not sure | Somewhat agree | Strongly agree | Main outcome | Missing |
| “Maintaining a healthy lifestyle reduces the risk of developing dementia” (unweighted) | 14 (0.9) | 91 (6.2) | 239 (16.2) | 723 (48.9) | 409 (27.7) | Agreeing N(%): 1132 (76.6) | 2 (0.1) |
| “Maintaining a healthy lifestyle reduces the risk of developing dementia” (weighted) | 16 (1.1) | 99 (6.7) | 245 (16.6) | 698 (47.2) | 419 (28.3) | Agreeing N(%): 1117 (75.5) | 1 (0.1) |
| “Changing lifestyle and health habits can help reduce my risk of developing dementia” (unweighted) | 13 (0.9) | 55 (3.7) | 257 (17.4) | 668 (45.2) | 482 (32.6) | Agreeing N(%): 1150 (77.8) | 3 (0.2) |
| “Changing lifestyle and health habits can help reduce my risk of developing dementia” (weighted) | 11 (0.8) | 61 (4.1) | 258 (17.4) | 658 (44.5) | 488 (33.0) | Agreeing N(%): 1146 (77.5) | 3 (0.2) |
| Statement on stroke N, (%) | Strongly disagree | Somewhat disagree | Not sure | Somewhat agree | Strongly agree |  | Missing |
| “Maintaining a healthy lifestyle does not reduce a person’s risk of stroke” (unweighted) | 594 (40.2) | 585 (39.6) | 160 (10.8) | 92 (6.2) | 47 (3.2) | Disagreeing N(%): 1179 (79.8) | 0 (0) |
| “Maintaining a healthy lifestyle does not reduce a person’s risk of stroke” (weighted) | 566 (38.3) | 561 (38.0) | 174 (11.8) | 115 (7.8) | 62 (4.2) | Disagreeing N(%): 1127 (76.3) | 0 (0) |
| “Changing lifestyle and health habits can help reduce my risk of having a stroke” (unweighted) | 6 (0.4) | 24 (1.6) | 79 (5.3) | 579 (39.2) | 788 (53.3) | Agreeing N(%): 1367 (92.5) | 2 (0.1) |
| “Changing lifestyle and health habits can help reduce my risk of having a stroke” (weighted) | 4 (0.3) | 31 (2.1) | 80 (5.4) | 606 (41.0) | 755 (51.1) | Agreeing N(%):  1361 (92.1) | 1 (0.1) |

**Legend.** Table S4 Perceptions on preventability of dementia and stroke weighted and unweighted.

Abbreviations: N: Number of participants. %: Percentage.

|  | **Table S5 Univariable logistic analysis** | | | | | | |
| --- | --- | --- | --- | --- | --- | --- | --- |
| **Dementia** | | | | | | | |
| maintaining a healthy lifestyle reduces the risk of developing dementia | |  | | **Agreeing N =** | **Proportion (95%CI)** | **Odds (95%CI)** | **P-value** |
|  |  | Age | >60 years (Ref) | 260 | 74.7 (70.1-79.3) | Ref |  |
|  |  |  | 40-60 | 265 | 72.9 (68.3-77.5) | 1.15 (0.85-1.54) | 0.361 |
|  |  |  | <40 | 580 | 77.2 ( 74.2-80.2) | 0.91 (0.65-1.27) | 0.579 |
|  |  | Sex assigned at birth | Male (Ref) | 582 | 80.8 (78.0-83.7) | Ref |  |
|  |  |  | Female | 516 | 70.4 (67.1-73.7) | 0.56 (0.43-0.72) | **<0.001** |
|  |  | Race/ethnicity | Non-Hispanic White (Ref) | 681 | 76.7 (73.9-79.5) | Ref |  |
|  |  |  | Non- Hispanic Black / African American | 126 | 71.2 (64.5-77.9) | 0.75 (0.52-1.07) | 0.115 |
|  |  |  | Asian | 56 | 79.4 (69.9-88.8) | 1.17 (0.64-2.12) | 0.612 |
|  |  |  | Hispanic / Latino | 215 | 77.7 (72.8-82.0) | 1.06 (0.76-1.47) | 0.75 |
|  |  |  | Other | 23 | 52.8 (38-67.6) | 0.34 (0.18-0.63) | **<0.001** |
|  |  | Education | Maximum high school (Ref) | 389 | 73.7 (69.5-77.1) | Ref |  |
|  |  |  | Associate degree | 551 | 76.6 (73.5-79.7) | 1.20 (0.93-1.55) | 0.171 |
|  |  |  | Graduate degree | 155 | 76.6 (70.8-82.4) | 1,20 (0.82-1.75) | 0.349 |
|  |  | Knowing someone with dementia or stroke | No (ref) | 212 | 72.5 (67.4-77.6) | Ref |  |
|  |  |  | Yes | 904 | 76.4 (74.0-78.8) | 1.23 (0.92-1.64) | 0.162 |
| Changing lifestyle and health habits can help reduce my risk of developing dementia | |  | | **Agreeing N =** | **Proportion (95%CI)** | **Odds (95%CI)** | **P value** |
|  |  | Age | >60 years (Ref) | 268 (76.7) | 76.7 (72.3-81.1) | Ref |  |
|  |  |  | 40-60 | 266 (73.1) | 73.1 (68.5-77.7) | 1.20 (0.89-1.64) | 0.234 |
|  |  |  | <40 | 599 (79.9) | 79.9 (77.0-82.7) | 0.82 (0.59-1.16) | 0.266 |
|  |  | Sex assigned at birth | Male (Ref) | 593 (82.3) | 82.3 (79.5-85.1) | Ref |  |
|  |  |  | Female | 534 (73.0) | 73.0 (69.8-76.2) | 0.58 (0.45-0.75) | **<0.001** |
|  |  | Race/ethnicity | Non-Hispanic White (Ref) | 687 (77.6) | 77.6 (74.9-80.3) | Ref |  |
|  |  |  | Non- Hispanic Black / African American | 131 (73.4) | 73.4 (66.9-79.8) | 0.80 (0.55-1.12) | 0.225 |
|  |  |  | Asian | 57 (79.2) | 79.2 (69.8-88.6) | 1.10 (0.61-1.98) | 0.761 |
|  |  |  | Hispanic / Latino | 225 (81.3) | 81.3 (76.7-85.9) | 1.25 (0.89-1.76) | 0.197 |
|  |  |  | Other | 29 (65.3) | 65.3 (46.6-84.0) | 0.54 (0.29-1.03) | 0.063 |
|  |  | Education | Maximum high school (Ref) | 382 (72.2) | 72.2 (67.8-76.6) | Ref |  |
|  |  |  | Associate degree | 586 (81.5) | 81.5 (78.4-84.6) | 1.70 (1.30-2.22) | **<0.001** |
|  |  |  | Graduate degree | 247 (76.6) | 76.6 (71.1-82.1) | 1.26 (0.86-1.83) | 0.236 |
|  |  | Knowing someone with dementia or stroke | No (ref) | 213 (73.3) | 73.3 (67.2-79.4) | Ref |  |
|  |  |  | Yes | 931 (78.7) | 78.7 (76.3-81.1) | 1.34 (1.00-1.80) | 0.051 |
|  | **Stroke** | | | | | | |
| Maintaining a healthy lifestyle does not reduce the risk of developing stroke | |  | | **Disagreeing N =** | **Proportion (95%CI)** | **Odds (95%CI)** | **P - value** |
|  |  | Age | >60 years (Ref) | 279 (80.1) | 80.1 (75.6-84.6) | Ref |  |
|  |  |  | 40-60 | 285 (78.2) | 78,.2 (73.5-82.9) | 0.89 (0.62-1.29) | 0.528 |
|  |  |  | <40 | 553 (73.6) | 73.6 (70.0-77.2) | 0.69 (0.51-0.94) | 0.019 |
|  |  | Sex assigned at birth | Male (Ref) | 571 (79.2) | 79.2 (75.9-82.5) | Ref |  |
|  |  |  | Female | 542 (73.9) | 73.9 (70.7-77.1) | 0.74 (0.58-0.95) | **0.017** |
|  |  | Race/ethnicity | Non-Hispanic White (Ref) | 712 (80.3) | 80.3 (77.7-82.9) | Ref |  |
|  |  |  | Non- Hispanic Black / African American | 127 (71.3) | 71.3 (64.7-77.9) | 0.61 (0.42-0.88) | **0.008** |
|  |  |  | Asian | 60 (83.8) | 83.8 (75.3-92.3) | 1.27 (0.66-2.44) | 0.468 |
|  |  |  | Hispanic / Latino | 197 (70.9) | 70.9 (65.6-76.2) | 0.60 (0.44-0.81) | **0.001** |
|  |  |  | Other | 17 (37.8) | 37.8 (23.6-52.0) | 0.15 (0.08-0.28) | **<0.001** |
|  |  | Education | Maximum high school (Ref) | 391 (73.8) | 73.8 (70.1-77.5) | Ref |  |
|  |  |  | Associate degree | 564 (78.4) | 78.4 (75.4-81.4) | 1.29 (0.99-1.68) | 0.057 |
|  |  |  | Graduate degree | 156 (76.5) | 76.5 (70.7-82.3) | 1.15 (0.79-1.68) | 0.456 |
|  |  | Knowing someone with dementia or stroke | No (ref) | 201 (68.8) | 68.8 (63.5-74.1) | Ref |  |
|  |  |  | Yes | 925 (78.1) | 78.1 (75.7-80.5) | 1.62 (1.22-2.15) | <**0.001** |
| Changing lifestyle and health habits can help reduce my risk of having a stroke | |  | | **Agreeing N =** | **Proportion (95%CI)** | **Odds (95%CI)** | **P - value** |
|  |  | Age | >60 years (Ref) | 339 (97.3) | 97.3 (95.6-99.0) | Ref |  |
|  |  |  | 40-60 | 337 (92.4) | 92.4 (89.7-95.1) | 0.33 (0.16-0.71) | **0.004** |
|  |  |  | <40 | 672 (89.5) | 89.5 (87.3-91.7) | 0.24 (0.12-0.47) | **<0.001** |
|  |  | Sex assigned at birth | Male (Ref) | 668 (92.5) | 92.5 (90.6-94.4) | Ref |  |
|  |  |  | Female | 673 (91.8) | 91.8 (91.3-94.7) | 0.91 (0.62-1.33) | 0.612 |
|  |  | Race/ethnicity | Non-Hispanic White (Ref) | 825 (93.0) | 93.0 (91.3-94.7) | Ref |  |
|  |  |  | Non- Hispanic Black / African American | 163 (92.2) | 92.2 (88.2-96.2) | 0.90 (0.49-1.64) | 0.720 |
|  |  |  | Asian | 62 (86.5) | 86.5 (78.6-99.4) | 0.49 (0.26-1.00) | 0.052 |
|  |  |  | Hispanic / Latino | 252 (91.0) | 91.0 (87.6-94.4) | 0.76 (0.47-1.24) | 0.276 |
|  |  |  | Other | 39 (88.6) | 88.6 (79.2-98.0) | 0.59 (0.22-1.54) | 0.279 |
|  |  | Education | Maximum high school (Ref) | 477 (89.9) | 89.9 (87.3-92.5) | Ref |  |
|  |  |  | Associate degree | 682 (94.9) | 94.9 (93.3-96.5) | 2.10 (1.40-3.26) | **<0.001** |
|  |  |  | Graduate degree | 180 (88.7) | 88.7 (84.3-93.1) | 0.88 (0.53-1.48) | 0.630 |
|  |  | Knowing someone with dementia or stroke | No (ref) | 254 (86.7) | 86.7 (82.8-90.6) | Ref |  |
|  |  |  | Yes | 1107 (93.5) | 93.5 (92.1-94.9) | 2.20 (1.46-3.32) | **<0.001** |

**Legend.** Table S5. Univariable logistic analysis for the four primary outcomes. The (Dis)agreeing column presents the percentage of the total participants that (dis)agreed with the statement for each variable. Abbreviations: Abbreviations: N: number of participants, Ref: reference, SD: standard deviation, % the percentage, CI confidence interval.

| Table S6. Multivariable logistic regression | | |  |
| --- | --- | --- | --- |
|  | Dementia | aOR (95% CI) | P-value |
| Knowing someone with dementia or stroke.  Knowing someone versus not (reference).  *Adjusted for age, sex assigned at birth, race/ethnicity, level of education, marital status , employment status, and being a caregiver for someone with dementia or stroke.* | Agreeing that maintaining a healthy lifestyle reduces the risk of developing dementia | 1.41 (1.10-1.96) | **0.045** |
|  | Agreeing that changing lifestyle and health habits can reduce their risk of developing dementia | 1.59 (1.14-2.24) | **0.007** |
|  | Stroke | aOR (95% CI) | P-Value |
|  | Disagreeing that maintaining a healthy lifestyle does not reduce the risk of developing stroke | 1.77 (1.27-2.47) | **<0.001** |
|  | Agreeing that changing lifestyle and health habits can reduce their risk of developing stroke | 2.31 (1.41-3.76) | **<0.001** |
| Sex assigned at birth.  Female versus male (reference).  *Adjusted for age, race/ethnicity, level of education, knowing someone with dementia or stroke, marital status , employment status and total personal income.* | Dementia | aOR (95%CI) | P-value |
|  | Agreeing that maintaining a healthy lifestyle reduces the risk of developing dementia | 0.59 (0.45-0.77) | **<0.001** |
|  | Agreeing that changing lifestyle and health habits can reduce their risk of developing dementia | 0.62 (0.47-0.82) | **<0.001** |
|  | Stroke | aOR (95%CI) | P-Value |
|  | Disagreeing that maintaining a healthy lifestyle does not reduce the risk of developing stroke | 0.76 (0.57-1.01) | 0.057 |
|  | Agreeing that changing lifestyle and health habits can reduce their risk of developing stroke | 0.83 (0.53-1.28) | 0.393 |

**Legend.** Table S6. Multivariable logistic regression analysis for the four primary outcomes. Abbreviations: aOR: adjusted Odds ratio. N: number of participants, Ref: reference, SD: standard deviation, % the percentage, CI confidence interval

| **Table S7 Stratification based on sex assigned at birth** | | | | |
| --- | --- | --- | --- | --- |
|  | | Female N (%) | Male N (%) | P-Value |
| Age | <40 | 351 (48.4) | 387 (54.0) | **0.017** |
|  | 40-60 | 180 (24.8) | 181 (181) |  |
|  | >60 | 196 (27.0) | 149 (20.8) |  |
| Race/Ethnicity | Non-Hispanic White | 460 (63.4) | 414 (58.3) | **0.002** |
|  | Non- Hispanic Black / African American | 89 (12.3) | 89 (12.5) |  |
|  | Asian | 32 (4.4) | 38 (5.4) |  |
|  | Hispanic / Latino | 115 (15.8) | 157 (22.1) |  |
|  | Other | 30 (4.1) | 12 (1.7) |  |
| Level of education | Max high school | 269 (37.3) | 252 (35.1) | 0.564 |
|  | Associate degree | 356 (49.4) | 358 (49.9) |  |
|  | Graduate degree | 96 (13.3) | 107 (14.9) |  |
| Knowing someone with dementia/stroke | Yes | 113 (15.4) | 173 (24.0) | **<0.001** |
|  | No | 620 (84.6) | 548 (76.0) |  |
| Marital status | Never married | 216 (30.5) | 263 (37.8) | **<0.001** |
|  | Currently married | 317 (44.7) | 358 (51.5) |  |
|  | Seperated,divorced or widowed | 176 (24.8) | 74 (10.6) |  |
| Employment | Employed | 371 (51.5) | 496 (70.3) | **<0.001** |
|  | Unemployed | 15 (2.1) | 24 (3.4) |  |
|  | Not in labor force | 335 (46.5) | 186 (26.3) |  |
| Personal Income | $ 0 - $ 10.275 | 206 (28.1) | 120 (16.7) | **<0.001** |
|  | $ 10.276 - $ 41.775 | 294 (40.1) | 207 (28.7) |  |
|  | $41.776 - $89.075 | 171 (23.3) | 257 (35.7) |  |
|  | >$ 89.076 | 62 (8.5) | 136 (18.9) |  |

**Legend.** Table S7: Stratification of baseline characteristics based on sex assigned at birth. Abbreviations:

N: Number of participants. %: Percentage.

| **Table S8. Sensitivity Analysis –**  difference between (i) ever knowing someone with dementia or stroke and (ii) ever caregiver for someone  with dementia or stroke with the four primary outcomes | | | | | |
| --- | --- | --- | --- | --- | --- |
| Outcome | Ever knowing someone with dementia or stroke | | Ever caregiver for someone with dementia or stroke | |  |
|  | N = | Proportion (95%CI) | N= | Proportion (95%CI) | P value |
| Dementia | | | | | |
| Agreeing that maintaining a healthy lifestyle reduces the risk of developing dementia | 904 | 76.4 (74.0-78.8) | 294 | 74.9 (70.6-79.2) | 0.284 |
| Agreeing that changing lifestyle and health habits can reduce risk of developing dementia | 931 | 78.7 (76.4-81.0) | 303 | 76.3 (72.1-80.5) | 0.540 |
| Stroke | | | | | |
| Disagreeing that maintaining a healthy lifestyle does not reduce a person’s risk of having a stroke | 925 | 78.1 (75.7-80.5) | 300 | 75.3 (71.1-79.5) | 0.948 |
| Agreeing that changing lifestyle and health habits can reduce risk of having a stroke | 1107 | 93.5 ( 92.1-94.9) | 369 | 92.7 (90.1-95.3) | 0.749 |

**Legend.** Table S8: Sensitivity analysis: difference between (i) ever knowing someone with dementia or

stroke and (ii) ever caregiver for someone with dementia or stroke with the four primary outcomes.

Abbreviations: N: Number of participants. 95%CI: 95 percent confidence interval

| Table S9. Sensitivity Analysis Multivariable logistic regression - Perceptions of preventability  Outcomes: Maintaining or changing lifestyle reduces dementia and stroke risk  Exposures: Ever knowing someone with dementia or stroke | | |  |
| --- | --- | --- | --- |
|  | Dementia | aOR (95% CI) | P-value |
| Knowing someone with dementia or stroke.  Knowing someone versus not (ref).  *Adjusted for age, sex assigned at birth, race/ethnicity, level of education, marital status, and being a caregiver for someone with dementia or stroke.* | Agreeing that maintaining a healthy lifestyle reduces the risk of developing dementia | 1.38 (1.00-1.92) | 0.053 |
|  | Agreeing that changing lifestyle and health habits can reduce risk of developing dementia | 1.55 (1.11-2.17) | **0.011** |
|  | Stroke | aOR (95% CI) | P-Value |
|  | Agreeing that maintaining a healthy lifestyle reduces the risk of developing stroke | 1.70 (1.22-2.37) | **0.002** |
|  | Agreeing that changing lifestyle and health habits can reduce risk of developing stroke | 2.11 (1.30-3.43) | **0.002** |

**Legend.** Table S9. Sensitivity Analysis: Multivariable logistic regression analysis for the four primary outcomes. Abbreviations: aOR: adjusted Odds ratio. N: number of participants, Ref: reference, SD: standard deviation, % the percentage, CI confidence interval

| Table S10 – Sensitivity analysis excluding “not sure” responses | | |  |
| --- | --- | --- | --- |
|  | Dementia | aOR (95% CI) | P-value |
| Knowing someone with dementia or stroke.  Knowing someone versus not (reference).  *Adjusted for age, sex assigned at birth, race/ethnicity, level of education, marital status , employment status, and being a caregiver for someone with dementia or stroke.* | Agreeing that maintaining a healthy lifestyle reduces the risk of developing dementia | 1.77 (1.09-2.88) | **0.020** |
|  | Agreeing that changing lifestyle and health habits can reduce their risk of developing dementia | 2.41 (1.28-4.55) | **0.006** |
|  | Stroke | aOR (95% CI) | P-Value |
|  | Disagreeing that maintaining a healthy lifestyle does not reduce the risk of developing stroke | 2.28 (1.48-3.50) | **<0.001** |
|  | Agreeing that changing lifestyle and health habits can reduce their risk of developing stroke | 2.61 (1.07-6.32) | **0.034** |

**Legend.** Table S10. Sensitivity Analysis: Multivariable logistic regression analysis for the four primary outcomes excluding “not sure” responses. Abbreviations: aOR: adjusted Odds ratio. N: number of participants, Ref: reference, SD: standard deviation, % the percentage, CI confidence interval
